# Multiplexed Droplet Digital PCR Assays for the Simultaneous Screening of Major Genetic Alterations in Tumors of the Central Nervous System

**DOI:** 10.3389/fonc.2020.579762

**Published:** 2020-11-12

**Authors:** Romain Appay, Frederic Fina, Doriane Barets, Catherine Gallardo, Isabelle Nanni-Metellus, Didier Scavarda, Daniel Henaff, Juline Vincent, Lise Grewis, Philippe Pourquier, Carole Colin, Dominique Figarella-Branger

**Affiliations:** ^1^ APHM, CHU Timone, Service d’Anatomie Pathologique et de Neuropathologie, Marseille, France; ^2^ Aix-Marseille Univ, CNRS, INP, Inst Neurophysiopathol, Marseille, France; ^3^ ID Solutions, Research and Development, Grabels, France; ^4^ APHM, CHU Nord, Service de Transfert d’Oncologie Biologique, Laboratoire de Biologie Médicale, Marseille, France; ^5^ APHM, CHU Timone, Service de Neurochirurgie pédiatrique, Marseille, France

**Keywords:** biomarkers, molecular screening test, multiplexed droplet digital PCR assay, glial and glioneuronal tumors, tumors of the central nervous system, formaldehyde-fixed sample tissue

## Abstract

The increased integration of molecular alterations to define tumor type or grade in central nervous system (CNS) tumor classification brings new challenges for the pathologist to make the best use of a precious limited tissue specimen for molecular studies. Within the different methods available to identify gene alterations, the droplet digital PCR (dPCR) constitutes a rapid, cost-effective, and very sensitive tool. In this study, we describe the development and validation of five multiplexed dPCR assays to detect major CNS biomarkers by using only small amounts of DNA extracted from formalin-fixed paraffin-embedded specimens. When compared to HRM-sequencing, NGS-sequencing, RNA-sequencing, or simplex digital PCR assays used as “gold standard” methods, these multiplexed dPCR assays displayed 100% specificity and sensitivity for the simultaneous detection of: 1/BRAF V600E mutation and KIAA1549:BRAF fusion; 2/FGFR1 N546K and K656E mutations and FGFR1 duplication; 3/H3F3A K27M and G34R/V mutations; 4/IDH1 R132X and IDH2 R172X mutations; and 5/TERT promoter mutations C228T and C250T. In light of the increased integration of molecular alteration, we believe that such strategies might help laboratories to optimize their screening strategies for routine diagnosis of pediatric and adult CNS tumors.

## Introduction

Recent progress in genome sequencing technologies and large-scale genomic studies has revolutionized our understanding of genetic alterations characteristics of many tumor types allowing more accurate tumor classification, which represents the basic concept established by the world health organization ‘‘WHO’’ classification of central nervous system (CNS) tumors in 2016 (1). Based on this updated classification, the diagnosis of several tumors requires the characterization of the underlying molecular abnormalities toward an “integrated” morphological and molecular diagnosis (*e.g.* glioblastoma, *IDH*-mutant). For other entities, although the diagnosis does not yet require the characterization of the underlying molecular abnormalities, it is nevertheless specified in the 2016 WHO classification that certain molecular alterations constitute a major additional putative diagnostic marker (*e.g.* highlighting a *KIAA1549:BRAF* fusion in pilocytic astrocytomas, as an example) (1).

This current “molecular diagnosis area” brings new challenges for the pathologist to make best use of a precious limited tissue specimen. Indeed, as the screening of relevant biomarkers is becoming increasingly important in the diagnostic process, or even sometimes mandatory, it requires access to high-performance and appropriate molecular tests in order to overcome analytical difficulties. Such investigations are inexorably costly, time-consuming, sample-consuming, and the sensitivity of various approaches might be insufficient in a context of a prominent background of non-tumoral DNA. These latter are particularly significant in the context of CNS neoplasm since the anatomic location is sometimes difficult to reach for the surgeon and thereby resulting in the examination of very small samples or in the tumor periphery with a low tumoral cellularity. Furthermore, several types of genetic alterations should be investigated, including mutations, fusions, or copy number variation, therefore multiplying the analytical chains (extraction of both DNA and RNA) and inevitably leading to the exhaustion of a valuable diagnostic material. Finally, frozen material may not always be available and DNA or RNA extracted from formalin-fixed paraffin-embedded samples (FFPEs) may be of limited use for molecular analyses due to chemical modifications of nucleic acids and degradation over time (2, 3).

Within the different methods available to identify gene alterations, the droplet digital PCR (dPCR) constitutes an interesting strategy as it is a rapid, cost-effective with low turnaround time, and a very sensitive tool. It is particularly useful on FFPE specimen since dPCR is a very robust approach on altered or fragmented DNA (4). Indeed, the detection and quantification of DNA copies are obtained by hydrolysis of a specific probes (Taqman), therefore requiring only small amplicons (70–100bp). In addition, dPCR limits the influence of enzymes inhibitors contained in formalin because their concentration is generally relatively low and their partitioning into only a few droplets has minimal influence on the analysis. Furthermore, it allows laboratories to equip themselves with a unique, readily available tool to screen for various types of molecular alterations including mutation and copy number variation with an absolute quantification for each alteration (5). Moreover, regarding fusions, we have previously demonstrated the high accuracy of dPCR to assess the presence of a *BRAF* duplication which is sufficient to predict a *KIAA1549:BRAF* fusion allowing a single analytical procedure limited to extracted DNA (6). However, the commonly used dPCR assays are target specific and do not cover multiple gene alterations. Thus, multiplexing dPCR assays might constitute a major additional advantage permitting the simultaneous detection of various genetic alterations and consequently tissue-saving material.

In this study, we developed and evaluated the efficacy of five multiplexed dPCR assays allowing the simultaneous detection of: 1/*BRAF* V600E mutation and *BRAF* exon 14 duplication associated with the *KIAA1549:BRAF* fusion; 2/*FGFR1* N546K and K656E mutations and *FGFR1* internal tandem duplication of the tyrosine-kinase domain; 3/*H3F3A* K27M and G34R/V mutations; 4/*IDH1* R132X and *IDH2* R172X mutations; and 5/*TERT* promoter (*pTERT*) C228T and C250T mutations.

## Material and Methods

### Low Grade Glial or Glioneuronal Tumor Cohort

We selected samples from 20 patients with a low grade glial or glioneuronal tumor for which one of the following alterations were previously known (**Supplementary Table 1**): five cases with *BRAF* V600E mutation, five cases with *KIAA1549:BRAF* fusion (including three with 16:9 fusion and two with 16:11 fusion), five cases with *FGFR1* N546K or K656E mutations (including one with N546K and four with K656E mutations), and five cases with *FGFR1* exon 16 duplication. These cases were retrieved from previously published cohorts (6, 7). *BRAF* and *FGFR1* mutations status were evaluated by HRM-sequencing as previously described (7) and were known for respectively 12/20 and 17/20 cases. Screening for *KIAA1549:BRAF* fusion was performed by RNA-sequencing analysis on frozen specimen as previously described (6), and the status was known for 15/20 cases. Finally, screening for *FGFR1* exon 16 duplication was performed by dPCR as previously described (7), and the status was known for 17/20 cases. Selected cases with known gene alteration were used to evaluate the BRAF and FGFR1 multiplex dPCR assay sensitivities, while negative cases for which the gene status was known were used to evaluate assay specificities.

### Diffuse Glial Tumor Cohort

The second cohort comprised samples from 40 French patients with a diagnosis of diffuse glioma for which *H3F3A*, *IDH1/2*, or *pTERT* gene alteration status was known (**Supplementary Table 1**). This included five cases with *H3F3A* K27M mutation, five cases with *H3F3A* G34R mutation, five cases with *IDH1* R132X mutation (including two R132H, one R132S, one R132C and one R132L), five cases with *IDH2* R172X mutation (including two R172K, one R172L, one R172M and one R172W), five cases with *pTERT* C228T mutation, and five cases with *pTERT* C250T mutation, and 10 cases without *IDH1/2*, *H3F3A* nor *pTERT* alteration. These alterations were evaluated by targeted HRM-sequencing (eight cases) or next generation sequencing (NGS, 32 cases) performed on formalin-fixed paraffin-embedded (FFPE) tissue sections. *H3F3A* mutation status was known for 39/40 cases, *IDH1/2* mutation status for 37/40 cases, and *pTERT* mutation status for 35/40 cases. It should be noted that four cases presented both *IDH2* and *pTERT* mutations. Selected cases with known gene alteration were used to evaluate the *H3F3A*, *IDH1/2*, and *pTERT* multiplex digital PCR assay sensitivities while negative cases for which the gene status was known were used to evaluate assays specificities.

### Genomic DNA Extraction

For the low grade CNS neoplasm cohort we retrieved the extracted DNA used for previously published analysis (6, 7). Regarding the high grade CNS neoplasm cohort, areas of viable and representative tumor were marked by a pathologist (DFB or RA). In all cases the percentage of tumor cells was above 80%. Then, tumor DNA was extracted from 4 × 5μm thick sections of FFPE tissue samples after dewaxing as previously published (8).

### Droplet Digital PCR Assays

Each multiplexed digital PCR (mdPCR) assays were developed in collaboration between APHM and ID-Solutions (Grabels, France). After analytical validation of each assay, they were produced and packaged as a commercial kit before being clinically validated. The aim was to validate each assay in its final version before its commercialization. To allow clinical validation, target positive controls (TPCs) were generated for each assay. TPCs allow the validation of the PCR workflow and the PCR mix, as they are composed of synthetical DNA harboring every alteration to be detected. These TPCs are also calibrated at low concentration with a known allele frequency or CNV (copy number variation). DPCR analyses were performed as previously described (7). Extracted DNA was quantified using the IDQUANTq kit (ID-Solutions) with the Magnetic Induction Cycler (Mic) PCR Machine Cycler from Bio Molecular Systems (Göttingen, Germany). After quantification, DNA concentration was adjusted. Eight microliters of DNA comprising 1 to 5 ng and 14 µl of PCR mix (ready to use) were used for each mdPCR assay. A similar amplification program (50°C 2 min; 95°C 10 min; 40 × 95°C 30 ss–60°C 1 min; 98°C 10 min) was used for all targets except for the pTERT multiplex which included 50 cycles instead of 40. Indeed, because of the high guanine-cytosine content in the TERT promoter region, amplifying these sequences required to increase the number of PCR cycles up to 50 in this assay for an unambiguous distinction of positive droplet clusters. The QX200 Droplet Digital PCR System (Bio-Rad) was used with the AutoDG droplet generator (Bio-Rad). Quantasoft Analysis Pro Software v1.0.596 (Bio-Rad) was used for the qualitative and quantitative analyses. The cut-off values of positive results for mutant detection are presented in **Supplementary Table 2**. Detection thresholds were set when the number of positives droplets was strictly above the limit of blank at 95% confidence interval defined for each assay depending on the number of replicates.

### Design of Multiplexed Droplet Digital PCR Assays

The dPCR partition samples into thousands of PCR reaction chambers with individual endpoint detection. Thus, different fluorescence profiles can be read and recorded individually and different strategies for multiplexing in dPCR have been developed in recent years (9). Our mdPCR assays were created by varying, for each target, the ratio of probes labeled with carboxyfluorescein (FAM) and/or hexachlorofluorescein (HEX) fluorophores, creating a specific fluorescence signature for each target. In order to simultaneously screen multiple gene alterations, we combined different multiplexing approaches and developed five different designs of mdPCR assays. Additionally, we added uracil N-glycosylase (UNG) in the PCR mixes in order to limit false positives mutants that could be linked to the formaline fixation inducing an incorporation of adenosines following the deamination of cytosines.

#### *BRAF* Multiplexed Droplet Digital PCR Assay

**Figure 1:**
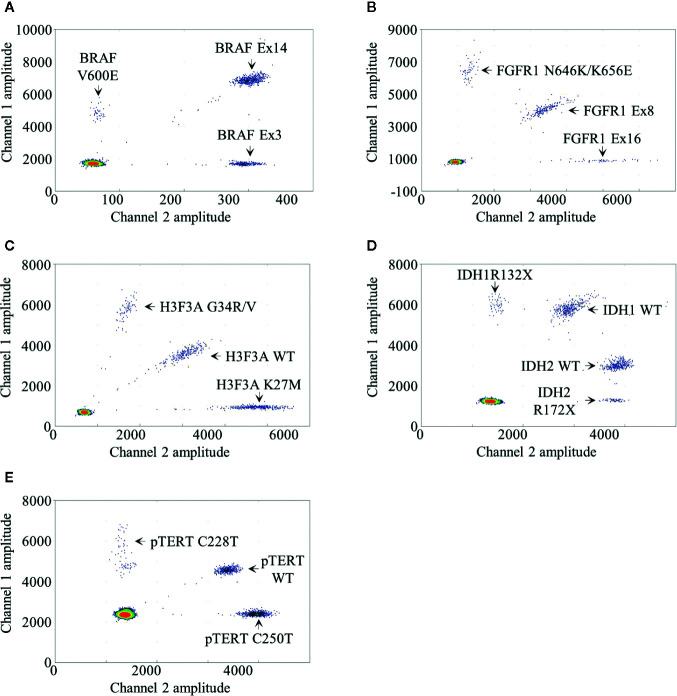
Droplet digital PCR results. Two dimensional representations of the BRAF **(A)**, FGFR1 **(B)**, H3F3A **(C)**, IDH1/2 **(D)** and pTERT **(E)** multiplexed droplet digital assays. Fluorescence amplitude at channels FAM and HEX are plotted at Y- and at X-axis, respectively. Each dot on the figure represents one droplet, and the corresponding targets are designated for each cluster. Bottom left clusters represent negative droplets.

We developed a BRAF mdPCR assay to screen simultaneously the presence of *BRAF* V600E mutation and *BRAF* exon 14 duplication, associated with the *KIAA1549:BRAF* fusion as previously published (6). In this assay, *BRAF* V600E mutation was targeted using 100% FAM labeled probe. BRAF exon 3, used as reference, was targeted using 100% HEX labeled probes and BRAF exon 14 was targeted using a mixture of both FAM and HEX labeled probes. This design resulted in a 2D plot with four clusters (**Figure 1A**): negative droplets, single positive droplets on FAM channel containing *BRAF* V600E mutant amplicons, single positive droplets on HEX channel containing the reference *BRAF* exon 3 amplicons and double positives droplets containing *BRAF* exon 14 amplicons.

Following the analysis and quantification of positive droplets on each channel, *BRAF* V600E fractional abundance (FA), equal to the ratio of mutant-DNA to wild-type DNA, was calculated as follows:

$$BRAF\;V600E\;FA\;(\%)=\frac{\left[BRAF\;V600E\;\right]}{\left[BRAF\;Exon\;3\right]}\times 100$$

The CNV represents for a haploid genome the ratio of target-DNA to reference-DNA multiplied by two. As previously published (6), a sample was considered duplicated if the CNV value was above 2.25 and the CNV min (calculated with a 95% confidence interval given by the Poisson distribution) was above 2.0, which means theoretically a monoallelic duplication in 25% of the analyzed cells. BRAF exon 14 CNV was calculated relative to exon 3 used as reference as follows:

$$BRAF\;Exon\;14\;CNV=\frac{\left[BRAF\;Exon\;14\right]}{\left[BRAF\;Exon\;3\right]}\times 2$$

#### *FGFR1* Multiplexed Droplet Digital PCR Assay

We developed a *FGFR1* mdPCR assay to screen simultaneously the presence of *FGFR1* N546K and K656E mutations as well as *FGFR1* exon 16 duplication. In this assay, *FGFR1* N546K and K656E mutations were targeted using 100% FAM labeled probe; *FGFR1* exon 16 was targeted using 100% HEX labeled probes; and *FGFR1* exon 8, used as reference, was targeted using a mixture of both FAM and HEX labelled probes. This design resulted in a 2D plot with four clusters (**Figure 1B**): negative droplets, single positive droplets on FAM channel containing *FGFR1* N546K or K656E mutant amplicons, single positive droplets on HEX channel containing *FGFR1* exon 16 amplicons, and double positive droplets containing the reference FGFR1 exon 8 amplicons. As previously detailed, *FGFR1* N546K/K656E FA was calculated as follows:

$$FGFR1\;\text{N}546\text{K}/K656E\;FA\;(\%)=\frac{\left[FGFR1\;\text{N}546\text{K}/\text{K}656\text{E}\right]}{\left[FGFR1\;Exon\;8\right]}\times 100$$

Similarly, FGFR1 exon 16 CNV was calculated relative to exon 8 used as reference as follows:

$$FGFR1\;Exon\;16\;CNV=\frac{\left[FGFR1\;Exon\;16\right]}{\left[FGFR1\;Exon\;8\right]}\times 2$$

#### *H3F3A* Multiplexed Droplet Digital PCR Assay

We developed a *H3F3A* mdPCR assay to screen simultaneously the presence of *H3F3A* G34R/V and K27M mutations. In this assay, two FAM labeled probes bound respectively the wild-type K27 locus sequence and the G34R/V mutations and two HEX labeled probes bound respectively the wild-type G34 locus and the K27M mutation, both within the same amplicon. This design resulted in a 2D plot with four clusters (**Figure 1C**): negative droplets, single positive droplets on FAM channel containing *H3F3A* G34R or G34V mutant amplicons, single positive droplets on HEX channel containing *H3F3A* K27M mutant amplicons and double positive droplets containing the *H3F3A* wild-type amplicons. *H3F3A* G34R/V or K27M FA was calculated as follows:

$$H3F3A\;G34R/V\;or\;K27M\;(\%)=\frac{\left[H3F3A\;G34\;R/V\;or\;\text{K}27\text{M}\right]}{\left[H3F3A\;G34\;R/V\;or\;\text{K}27\text{M] }+\text{ [}H3F3A\;WT\right]}\times 100$$

#### *IDH1/2* Multiplexed Droplet Digital PCR Assay

We developed an *IDH1/2* mdPCR assay to screen simultaneously the presence of *IDH1* R132X and *IDH2* R172X mutations. In this Drop-Off Assay (10), within the same *IDH1* or *IDH2* amplicons, one probe bound a “reference” sequence distant from the hotspot mutation target site and a second Drop- off probe bound at the hotspot mutation target site (R132 or R172). Thus, one FAM-labeled probe bound a “reference” sequence distant from the *IDH1* R132 locus and a second Drop-off HEX-labeled probe bound the R132 wild-type locus, both within the same amplicon. In addition, one HEX labeled probe bound a “reference” sequence distant from the *IDH2* R172 locus and a second Drop-off FAM-labeled probe bound the R172 wild-type locus both within the same amplicon. Finally, the separation of *IDH1* and *IDH2* wild-type clusters was achieved by varying ratio of FAM and HEX labeled probes. This design resulted in a 2D plot with five clusters (**Figure 1D**): negative droplets, single positive droplets on FAM channel containing *IDH1* R132X mutant amplicons, single positive droplets on HEX channel containing *IDH2* R172X mutant amplicons and two clusters of double positive droplets with different ratios containing the *IDH1* and *IDH2* wild-type amplicons. *IDH1* R132X or *IDH2* R172X FA was calculated as follows:

$$IDH1\;R132X\;FA\;(\%)=\frac{\left[IDH1\;R132X\;\right]}{\left[IDH1\;R132X\;\text{] }+\text{ [}IDH1\;WT\right]}\times 100$$

$$IDH2\;R172X\;FA\;(\%)=\frac{\left[IDH2\;R172X\;\right]}{\left[IDH2\;R172X\;\text{] }+\text{ [}IDH2\;WT\right]}\times 100$$

#### *pTERT* Multiplexed Droplet Digital PCR Assay

We developed a *pTERT* mdPCR assay to screen simultaneously the presence of *pTERT* C228T and C250T mutations. In this assay, two FAM-labeled probes bound respectively the wild-type C228 locus sequence and the C250T mutation and two HEX-labeled probes bound respectively the wild-type C250 locus sequence and the C228T mutation, both within the same amplicon. This design resulted in a 2D plot with four clusters (**Figure 1E**): negative droplets, single positive droplets on FAM channel containing *pTERT* C228T mutant amplicons, single positive droplets on HEX channel containing *pTERT* C250T mutant amplicons and double positive droplets containing the *pTERT* wild-type amplicons. *pTERT* C228T or C250T FA were calculated as follows:

$$pTERT\;C228T\;or\;C250T\;FA\;(\%)=\frac{\left[pTERT\;C228T\;or\;C250T\right]}{\left[pTERT\;C228T\;or\;C250T\text{] }+\text{ [}pTERT\;WT\right]}\times 100$$

## Results

In this study we aimed to set up five mdPCR assays to simultaneously screen multiple gene alterations on DNA extracted from FFPE tissue samples. To achieve this goal, we evaluated *BRAF* and *FGFR1* mdPCR assays in low grade glial or glioneuronal tumors and *H3F3A*, *IDH1/2* and *pTERT* mdPCR assays in diffuse gliomas. We used RNA-sequencing, HRM-sequencing, NGS or dPCR as gold standard methods to calculate the sensitivity and specificity of each mdPCR assay (**Table 1**). Despite the presence of UNG in all mdPCR assays to limit the impact of formaldehyde on DNA extracted from FFPE samples, we observed that some residual DNA sequences may be falsely detected as positive due to the high dPCR sensitivity. Therefore, a cut-off value of positive result >1% was used to consider a sample as mutated.

**Table 1:** Results of the multiplexed digital PCR assays compared to reference methods.

	mdPCR/Reference Method	mdPCR Sensitivity	mDPCR Specificity
***BRAF* Multiplex**			
*BRAF* V600E mutation	5/5	1.00	1.00
*BRAF* wildtype	7/7		
*KIAA1549:BRAF* fusion	5/5		
No *BRAF* fusion	10/10		
***FGFR1* Multiplex**			
*FGFR1* N546K or K656E mutations	5/5	1.00	1.00
*FGFR1* wildtype	12/12		
*FGFR1* ex. 16 duplication	5/5		
No *FGFR1* duplication	12/12		
***H3F3A* Multiplex**			
*H3F3A* K27M mutation	5/5	1.00	1.00
*H3F3A* G34R/V mutation	5/5		
*H3F3A* wildtype	29/29		
***IDH1/2* Multiplex**			
*IDH1* R132X mutation	5/5	1.00	1.00
*IDH2* R172X mutation	6/6		
*IDH1/2* wildtype	24/24		
***pTERT* Multiplex**			
*pTERT* C228T mutation	8/8	1.00	1.00
*pTERT* C250T mutation	5/5		
*pTERT* wildtype	21/21		

### Characteristics of the Cohorts

As previously mentioned, all cases were selected on the basis of a previously known gene alteration, and two different cohorts were designed. The low grade glial or glioneuronal tumor cohort comprised samples with *BRAF* or *FGFR1* gene alterations and included 11 dysembryoplastic neuroepithelial tumors, seven pilocytic astrocytomas, and two gangliogliomas. On the other hand, the diffuse glioma cohort comprised samples with *H3F3A*, *IDH1/2*, and/or *pTERT* gene alterations and included five diffuse midline gliomas *H3F3A* K27M-mutant, four anaplastic oligodendrogliomas *IDH*-mutant and 1p/19q-codeleted, three anaplastic astrocytomas *IDH*-mutant, four glioblastomas *IDH*-mutant, and 24 glioblastomas *IDH*-wildtype. The characteristics of the cohorts are presented in **Supplementary Table 1**.

### Evaluation of BRAF and FGFR1 Multiplexed Droplet Digital PCR Assay Among Low Grade Glial or Glioneuronal Tumors

Among the low grade glial or glioneuronal tumor cohort, all selected cases with *BRAF* V600E mutation (5/5) or *FGFR1* N546K or K656E mutation (5/5) evaluated by HRM-sequencing were also detected with the mdPCR assays. Mutant allele frequencies ranged from 3.8 to 26.1% (**Supplementary Table 1**). It is worth noticing that one case with a *FGFR1* G539R mutation was not detected by the *FGFR1* mdPCR assay. All cases without *BRAF* V600E mutation (7/7) or *FGFR1* N546K or K656E mutation (12/12) were also negative with the mdPCR assays. These included two cases with BRAF V600E mutant allele frequency of 0.3 and 0.1% and thus considered as negative.

In addition, all selected cases with already known *KIAA1549:BRAF* fusion evaluated by RNA-sequencing were also positive with the *BRAF* mdPCR assay (5/5). Similarly, all cases with known *FGFR1* duplication evaluated by dPCR were also detected with the *FGFR1* mdPCR assay (5/5). Cases without *KIAA1549:BRAF* fusion (10/10) or *FGFR1* duplication (12/12) were also negative with the mdPCR assays.

Overall, in comparison with the results previously obtained by HRM-sequencing and RNA-sequencing as the gold standard methods, the *BRAF* and *FGFR1* mdPCR assays had a sensitivity and specificity of 100% (**Table 1**).

### Evaluation of H3F3A, IDH1/2, and pTERT Multiplexed Droplet Digital PCR Assays Among Diffuse Gliomas

Among the diffuse glioma cohort, all selected cases with known *H3F3A* mutation (10/10), *IDH1*, or *IDH2* mutation (11/11) or *pTERT* mutation (13/13) evaluated by HRM-sequencing or NGS were also positive with the mdPCR assays. The detected alterations included: five *H3F3A* K27M mutations, five *H3F3A* G34R, two *IDH1* R132H, one *IDH1* R132C, one *IDH1* R132S, one *IDH1* R132L, three *IDH2* R172K, one *IDH2* R172L, one *IDH2* R172M, one *IDH2* R172W, eight *pTERT* C228T, and five *pTERT* C250T mutations. All cases without *H3F3A* K27M or G34R mutation (29/29), *IDH1* R132X or *IDH2* R172X mutation (24/24) or *pTERT* C228T or C250T mutation (21/21) were also negative with the mdPCR assays. These included one case with H3F3A K27M mutant allele frequency of 0.04% and one case with IDH1 R132X mutant allele frequency of 0.6% and thus both considered as negative. Unfortunately, the IDH1/2 and pTERT mdPCR assays could not be performed for respectively two and one cases because all the sample and extracted DNA had been used.

Overall, in comparison with the results obtained by HRM-sequencing or NGS as gold standard methods, the *H3F3A*, *IDH1/2*, and *pTERT* mdPCR assays had a sensitivity and specificity of 100% (**Table 1**).

## Discussion

In light of the increased integration of molecular alterations to define tumor type or grade, especially in CNS tumor classification, the need to develop appropriate molecular tests is becoming increasingly important. In this study, we describe the development and validation of five multiplexed digital PCR assays to sensitively and rapidly detect several clinically useful genetic alterations by using small amounts of DNA extracted from FFPE specimens. When compared to HRM-sequencing, NGS, RNA-sequencing or simplex digital PCR assays used as “gold standard” methods, these mdPCR assays displayed 100% specificity and sensitivity for the simultaneous detection of: 1/*BRAF* V600E mutation and *KIAA1549:BRAF* fusion; 2/*FGFR1* N546K and K656E mutations and *FGFR1* duplication; 3/*H3F3A* K27M and G34R/V mutations; 4/*IDH1* R132 and *IDH2* R172 mutations; and 5/*TERT* promoter mutations C228T and C250T. We believe that such mdPCR approach might be particularly useful in routine practice since an accurate diagnosis is obviously mandatory and the genetic alterations targeted by these mdPCR assays represent major molecular markers for glioma classification.

The *BRAF* and *FGFR1* mdPCR assays might particularly be useful in the context of pediatric-type glial or glioneuronal tumors. Indeed, this is a heterogeneous group of tumors which are often challenging to diagnose because of overlapping pathological criteria. Nevertheless, most of these tumors appear to be driven by a characteristic single driver genetic alteration, commonly affecting the MAPK pathway (11, 12). Although the characterization of specific molecular abnormalities is not yet mandatory according to the WHO 2016 classification (1), they constitute a major support to the diagnostic process and might be required in future classifications. Indeed, the cIMPACT-NOW working group (the Consortium to Inform Molecular and Practical Approaches to CNS Tumor Taxonomy), established in 2016 to propose new recommendations between WHO updates (13), has recently reviewed the status of the diffuse pediatric gliomas (14). The consortium recommends using an integrated diagnosis combining histological characteristics with the presence of genetic alterations including the *BRAF* V600E mutation, the *FGFR1* mutations or duplication, other MAPK pathway alterations or *MYB/MYBL1* alterations (14). The designed and validated mdPCR assays allow screening easily for most of these alterations. We previously demonstrated that the detection of BRAF exon 14 duplication was sufficient to predict the different types of *KIAA1549* and *BRAF* gene fusion (6). The most common being a fusion between exon 16 of *KIAA1549* and exon 9 of *BRAF* and less frequent variants include fusion between *KIAA1549* exon 15 and *BRAF* exon 9 or *KIAA1549* exon 16 and *BRAF* exon 11 (15). Furthermore, we showed that 1 ng of amplifiable DNA is sufficient to detect the *KIAA1549:BRAF* fusion if this alteration is present in at least 25% of the total amounts of analyzed cells. It is nevertheless important to note that the presented BRAF multiplexed assay is not designed to detect other rare fusions involving *BRAF* that have been reported in pilocytic astrocytomas including *FAM131:BRAF*, *RNF130:BRAF*, *CLCN6:BRAF*, *MKRN1:BRAF*, *GNAI1:BRAF*, and *GTF2I:BRAF* (12, 16–18). Another mdPCR assay targeting *PIK3CA* mutations, which also constitute a common genetic alteration of pediatrics glial and glioneuronal tumors, has also been designed. Nevertheless, the results are not presented in this study since we are currently validating its sensitivity and specificity. Unfortunately, we failed in designing a mdPCR assay that would allow the simultaneous detection of *MYB* and *MYBL1* alterations due to the wide variety of rearrangements reported (19).

The *H3F3A*, *IDH1/2*, and *pTERT* multiplexed dPCR assays might be particularly useful in the context of high grade diffuse gliomas for which the screening of the targeted molecular alteration is mandatory for an accurate diagnosis. Indeed, the evidence of the *H3F3A* K27M mutation is required to retain a diagnosis of diffuse midline glioma (1). Although not yet included in the WHO 2016 classification, gliomas harboring the *H3F3A* G34R/V mutation will be recognized as a new entity in the future WHO classification as recommended by the cIMPACT-NOW consortium (20). It is to note that even though the designed mdPCR assay successfully detected the G34V mutation on artificial sequences, we could not validate the method on human sample as this mutation is uncommon and we were not able to identify a patient with a G34V alteration in our database. The well-known *IDH1/2* mutations are required to discriminate *IDH*-mutant from *IDH*-wild-type gliomas which constitute the most common primary adult brain tumors. Finally, in the context of *IDH*-wild-type glioma, the evidence of a *pTERT* mutation is sufficient to retain the diagnosis of *IDH*-wild-type glioblastoma grade IV even in the absence of microvascular proliferation or necrosis according to the recommendations of the c-IMPACT-NOW group (21).

When compared to other approaches, the dPCR represents an easy-to-use, rapid and cost effective tool which can be performed using only very small amounts of DNA extracted from FFPE tissue samples (**Table 2**). Indeed, multiplexed dPCR assay demonstrates better performance on FFPE with reduced technical time and equipment cost when compared to conventional approaches such as targeted Sanger sequencing or next generation sequencing or RNAseq. In this study, the mdPCR techniques were successful on all samples with only 1 to 5 ng of DNA extracted from up to 20-year-old FFPE specimens (**Supplementary Table 1**). Furthermore, such multiplexed dPCR assays improve the analytical chain by allowing the simultaneous detection of different types of alterations with a single DNA extraction. This is particularly useful in the context of pediatric-type glial or glioneuronal tumors as multiplexed dPCR assays allows the simultaneous detection of mutations and copy number variation or fusion. Such cases would require both DNA extraction for mutation evaluation with NGS or Sanger sequencing and RNA extraction or additional tissue section for CNV or fusion evaluation performed respectively with RNAseq or FISH analysis. Additionally, we previously reported its usefulness in a prospective diagnostic routine (6).

**Table 2:** Comparison between the techniques commonly used for the detection of targeted alterations.

	FISH	RNAseq fusion panel	NGS mutation panel	Sanger	DNA-Methylation profiling	Multiplexed dPCR
**Amount and type of material**	Very low (tissue section)	Moderate (100–200 ng of RNA)	Low (5–10 ng of DNA)	Moderate (10 ng of DNA)	High (250–500 ng of DNA)	Very low (1–5 ng of DNA)
**Detected alterations**	Fusions	Fusions and duplications	Mutations and copy number variations	Mutations	*Tumor class*	Fusions, duplications and mutations
**Number of alterations detected simultaneously**	One	Many	Many	Few	N/A	Few
**Performance on FFPE material**	Low to Moderate	Moderate	Moderate	Moderate	Moderate	High
**Sensitivity on FFPE material**	Operator-dependent	Depending on level of expression of fusion/duplication	1 to 5%	10 to 20%	N/A	1%
**Time from pre-analytic to post-analytic (days)**	2	4	4	3	5	2
**Technical resources and cost**	Very low	High	High	Moderate	High	Low
**Technical complexity**	Low	High	High	High	High	Moderate

In this study, we used the Droplet Reader QX200TM (Bio-Rad) which analysis is limited to two fluorescence reading channels (FAM and HEX). Different approaches can be applied for multiplexing dPCR (22). The ratio-based strategy, used in our study, is based on the targeting of each target with different ratios of FAM and HEX-labeled probes resulting in the separation of droplet clusters (it requires two probes with different fluorophores for each target). A second amplitude-based strategy might also be used. The principle is to perform dPCR reaction with different concentrations of probes depending on the target resulting in droplet cluster separation. Both of these strategies may be combined to further increase the number of targeted alterations in a single assay. However these multiplexed strategies come with the challenge of accurate separation of fluorescent signals. Indeed, due to the action of formaldehyde on FFPE tissues, the presence of localized inhibitor in some reaction chambers can cause a decrease in signal, resulting in a “rain” of droplets (presence of positives droplets below the main cluster). Thus, positive droplets might fall in a cluster attributed to another alteration resulting in false positive in the latter. For this reason, the screening capabilities of these mdPCR assays are still limited to few alterations and analysis of a large panel of targets is difficult.

Because of the multiplexing limitations of digital PCR, other approaches for molecular screening may have specific advantages depending on the context (**Table 2**). As an example, NGS allows the simultaneous identification of a broad range of mutations and CNV. It can be cost-effective for an individual sample for which a large panel of biomarkers needs to be investigated. DNA-methylation profiling is also highly accurate to classify CNS tumors but it remains expensive and tissue-consuming. Moreover for some tumor class, it does not provide the underlying genetic alteration (23). Therefore, multiplexed dPCR assays would be specifically appropriate for histomolecular classification of gliomas and glioneuronal tumors as most laboratories are likely to have the capacities to use dPCR technology when compared to NGS. Furthermore, dPCR can also be used in many applications requiring high sensitivity, particularly for the detection of circulating tumor DNA in blood plasma or cerebrospinal fluid (24).

## Conclusion

The present study showed the usefulness of five multiplexed digital PCR assays to quickly, easily and simultaneously assess the status of several major CNS biomarkers by using small amounts of DNA extracted from FFPE tissue. We hope that such strategies might help laboratories to optimize their screening strategies for routine diagnosis of pediatric and adult CNS tumors.

## Data Availability Statement

The original contributions presented in the study are included in the article/**Supplementary Material**. Further inquiries can be directed to the corresponding author.

## Ethics Statement

The studies involving human participants were reviewed and approved by the APHM ethics committee. Written informed consent to participate in this study was provided by the participants’ legal guardian/next of kin.

## Author Contributions

RA, DF-B, and CC supervised the study and wrote the manuscript. DS contributed to build the tissue cohorts. DF-B and RA made the selection of the cohorts (FFPE material) and performed pathological review. FF, DH, JV, LG, and PP designed the multiplexed droplet digital PCR assays. IN-M performed genomic DNA extraction. FF, DB, and CG performed the multiplexed droplet digital PCR assays and contributed to data analysis. All authors contributed to the article and approved the submitted version.

## Funding

We thank the ARTC-Sud patients’ association (Association pour le Recherche sur les Tumeurs Cérébrales), the Association Cassandra, the Imagine For Margo Association, the SFCE (*Société Française de Lutte contre les Cancers et Leucémies de l’Enfant et de l’Adolescent*) the Cancéropôle PACA, and the GIRCI Méditerranée (GlioMark protocol) for their financial support.

## Conflict of Interest

FF, DH, JV, LG, and PP are all members of Id-Solutions, Grabels, France.

The remaining authors declare that the research was conducted in the absence of any commercial or financial relationships that could be construed as a potential conflict of interest.
