# Supplementary material for: Multiplexed Droplet Digital PCR Assays for the Simultaneous Screening of Major Genetic Alterations in Tumors of the Central Nervous System
Source: Front Oncol. 2020 Nov 12;10:579762. doi: 10.3389/fonc.2020.579762 (PMC7689380; doi:10.3389/fonc.2020.579762)
Supplement: Supplementary file 2 [file Table_2.docx]

**Supplementary Table 2** Limit of blank and limit of detection for each targets

| **Targets** | **Number of replicates** | **Limit of Blank at 95% confidence interval**  **(Total positive droplets)** | **Limit of detection**  **(Total positive droplets)** |
| --- | --- | --- | --- |
| **BRAF V600E** | Single | 3 | 4 |
|  | Duplicate | 4 | 5 |
|  | Triplicate | 4 | 5 |
| **FGFR1 N546K or K656E** | Single | 0 | 2 |
|  | Duplicate | 0 | 2 |
|  | Triplicate | 0 | 2 |
| **H3F3A K27M** | Single | 0 | 2 |
|  | Duplicate | 0 | 2 |
|  | Triplicate | 0 | 2 |
| **H3F3A G34R/V** | Single | 2 | 3 |
|  | Duplicate | 3 | 4 |
|  | Triplicate | 3 | 4 |
| **IDH1 R132X** | Single | 3 | 4 |
|  | Duplicate | 4 | 5 |
|  | Triplicate | 5 | 6 |
| **IDH2 R172X** | Single | 4 | 5 |
|  | Duplicate | 5 | 6 |
|  | Triplicate | 6 | 7 |
| **pTERT C228T** | Single | 4 | 5 |
|  | Duplicate | 6 | 7 |
|  | Triplicate | 7 | 8 |
| **pTERT C250T** | Single | 5 | 6 |
|  | Duplicate | 7 | 8 |
|  | Triplicate | 9 | 10 |
